# Listeria monocytogenes Requires the RsbX Protein To Prevent SigB Activation under Nonstressed Conditions

**DOI:** 10.1128/JB.00486-21

**Published:** 2022-01-18

**Authors:** Ana H. Oliveira, Teresa Tiensuu, Duarte N. Guerreiro, Hasan Tükenmez, Charlotte Dessaux, Francisco García-del Portillo, Conor O’Byrne, Jörgen Johansson

**Affiliations:** a Laboratory for Molecular Infection Medicine Sweden, Umeå University, Umeå, Sweden; b Department of Molecular Biology, Umeå University, Umeå, Sweden; c Umeå Centre of Microbial Research, Umeå University, Umeå, Sweden; d Bacterial Stress Response Group, Microbiology, School of Natural Sciences, National University of Ireland, Galway, Ireland; e Department of Chemistry, Umeå University, Sweden; f Laboratory of Intracellular Bacterial Pathogens, National Center of Biotechnology, (CNB)-CSIC, Spain; Queen Mary University of London

**Keywords:** *Listeria monocytogenes*, RsbX, SigB, stressosome, acid stress, motility, flagellar motility

## Abstract

The survival of microbial cells under changing environmental conditions requires an efficient reprogramming of transcription, often mediated by alternative sigma factors. The Gram-positive human pathogen Listeria monocytogenes senses and responds to environmental stress mainly through the alternative sigma factor σ^B^ (SigB), which controls expression of the general stress response regulon. SigB activation is achieved through a complex series of phosphorylation/dephosphorylation events culminating in the release of SigB from its anti-sigma factor RsbW. At the top of the signal transduction pathway lies a large multiprotein complex known as the stressosome that is believed to act as a sensory hub for stresses. Following signal detection, stressosome proteins become phosphorylated. Resetting of the stressosome is hypothesized to be exerted by a putative phosphatase, RsbX, which presumably removes phosphate groups from stressosome proteins poststress. We addressed the role of the RsbX protein in modulating the activity of the stressosome and consequently regulating SigB activity in L. monocytogenes. We show that RsbX is required to reduce SigB activation levels under nonstress conditions and that it is required for appropriate SigB-mediated stress adaptation. A strain lacking RsbX displayed impaired motility and biofilm formation and also an increased survival at low pH. Our results could suggest that absence of RsbX alters the multiprotein composition of the stressosome without dramatically affecting its phosphorylation status. Overall, the data show that RsbX plays a critical role in modulating the signal transduction pathway by blocking SigB activation under nonstressed conditions.

**IMPORTANCE** Pathogenic bacteria need to sense and respond to stresses to survive harsh environments and also to turn off the response when no longer facing stress. Activity of the stress sigma factor SigB in the human pathogen Listeria monocytogenes is controlled by a hierarchic system having a large stress-sensing multiprotein complex known as the stressosome at the top. Following stress exposure, proteins in the stressosome become phosphorylated, leading to SigB activation. We have studied the role of a putative phosphatase, RsbX, which is hypothesized to dephosphorylate stressosome proteins. RsbX is critical not only to switch off the stress response poststress but also to keep the activity of SigB low at nonstressed conditions to prevent unnecessary gene expression and save energy.

## INTRODUCTION

Listeria monocytogenes is a Gram-positive bacterium causing listeriosis, a life-threatening infection acquired from the ingestion of contaminated food. Listeriosis is particularly dangerous in certain high-risk groups, which include children, pregnant women, the elderly, and immunocompromised people, having a mortality rate between 20 and 30% (1). Despite being mainly a soil bacterium, it is commonly found in a wide range of different environments (e.g., water, animal feces, decaying vegetation, and food destined for human consumption). This ubiquity can be largely explained by the number of stress adaptations this bacterium has evolved to survive a variety of harsh environments, including those encountered during its transition from a saprophytic life to one within the human body (2–4).

The challenges presented by the host during infection can be overcome by L. monocytogenes using a variety of adaptive mechanisms (2, 5). For instance, the bacterium can utilize glutamate decarboxylation and arginine deamination to control its cytoplasmic pH, use bile salt hydrolase and drug efflux pumps to protect against bile stress, and use solute transport systems for osmoregulation when facing osmotic stress (3, 6). It can also deploy a well-coordinated repertoire of virulence factors (2). Its ability to enter epithelial cells in the intestine is due to the use of adhesins, like internalin A and B (InlA and InlB, respectively), that have specific receptors on the host cell surface (E-cadherin and c-Met, respectively), allowing the internalization of the bacterium. Listeriolysin O (LLO) and the phospholipases PlcA and PlcB assist L. monocytogenes in lysing and escaping the vacuole. Once in the cytosol of the host, it utilizes phosphorylated sugars, allowing rapid bacterial replication. Bacterial spread from cell to cell is mediated by ActA, a bacterial protein recruiting the Arp2/3 complex of the host cell, which, in turn, promotes actin polymerization and bacterial propulsion, eventually allowing invasion into an adjacent cell. Many of these events are under the control of the master transcriptional regulator of virulence, PrfA (positive regulatory factor A). Therefore, an efficient transcriptional response plays a key role in the survival of this pathogen in different environmental pressures (7, 8).

Bacterial sigma factors are important for stress adaptation since they determine which set of genes are transcribed at specific conditions. They interact with the RNA polymerase and direct the complex to specific promoter sequences upstream of target operons. The alternative stress sigma factor B (SigB) is one of the key components in the general stress response of several Gram-positive bacteria, controlling the transcription of a large number of stress-related genes (reviewed in reference 9). SigB was identified in L. monocytogenes based on its homology to SigB of the closely related nonpathogenic bacterium Bacillus subtilis (10, 11). In L. monocytogenes, SigB controls the response to several stress cues, such as osmotic stress (10, 12–14), light (4, 15–17), and acid stress (11, 18), and also virulence and central metabolism (19–21).

The activation of SigB occurs through a complex system of anti-sigma and anti-anti-sigma factors (22). Environmental signals are supposedly sensed and integrated into the regulatory pathway by a large multiprotein complex known as the stressosome (23, 24). In L. monocytogenes, this structure is formed by several proteins; RsbS and RsbT form the stressosome core, and RsbR1 and its paralogs RsbL (Lmo0799), RsbR2 (Lmo0161), RsbR3 (Lmo1642), and RsbR4 (Lmo1842) have been proposed to act as stress sensors. The stress sensor proteins expose their N-terminal domains as protrusions on the surface of the stressosome (25, 26). The N termini are thought to function as sensory domains that integrate the different stress signals and allow the activation of SigB. Despite much investigation, the molecular details underlying stress sensing in the RsbR1 and its paralogs remain unknown except to some extent for light sensing. Blue light stress has been proved to be sensed through the stressosome by the blue light receptor RsbL, ultimately resulting in activation of SigB (4, 15–17).

The current model accounting for SigB activation in L. monocytogenes is mostly based on data obtained in B. subtilis: When exposed to stress, the RsbR1 and RsbS proteins in the stressosome are phosphorylated by the protein kinase RsbT, ultimately leading to the release of RsbT from the stressosome. Liberated RsbT can interact with RsbU, promoting its phosphatase activity, prompting removal of a phosphate group from RsbV (anti-anti-sigma protein). Dephosphorylated RsbV is then able to bind RsbW (anti-sigma factor), leading to the release of SigB, which can then interact with the RNA polymerase and induce transcription of stress response genes. This partner-switching mechanism is controlled by the phosphorylation state of RsbV, which is regulated by the actions of the RsbU phosphatase and the RsbW kinase. When the bacteria are not experiencing significant stress, RsbV exists predominantly in the phosphorylated form, which allows the interaction between SigB and RsbW and consequently blocks SigB activation. Although stress-mediated activation of SigB is likely to be similar between *Listeria* and B. subtilis, differences exist. For instance, the four RsbR1 paralogues share very low sequence identity at their N-terminal domains compared to the RsbR1 paralogues of B. subtilis. In B. subtilis, energy deprivation can induce SigB activation by a mechanism not present in L. monocytogenes, but expressing RsbR1_Lm_ in B. subtilis allows the *Bacillus* stressosome to detect nutritional starvation (27). Since B. subtilis is a spore-forming bacterium, its response to stress could be calibrated somewhat differently than for L. monocytogenes (a nonspore-forming bacterium). However, similarities are also identified: despite not being a pathogenic bacterium *per se*, B. subtilis can also encounter the gastrointestinal (GI) tract by consumption of certain fermented products. In addition, closely related *Bacillus* species (e.g., B. cereus) that also possess SigB are foodborne pathogens that cause infections in the GI tract (28). Overall, this emphasizes that further understanding of stress-induced SigB activation in L. monocytogenes is needed.

**FIG 1:**
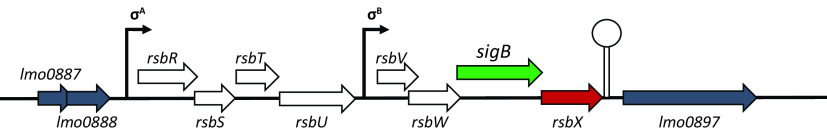
Schematic representation of the *sigB* locus in L. monocytogenes.

The details of the signal transduction pathway leading to SigB activation in L. monocytogenes, as well as the stressosome structure, are now becoming better understood (2, 14, 20, 25, 26, 29, 30). Having SigB constitutively active is, however, costly, making the bacterium less competitive, as has been observed for a strain lacking RsbX (30). RsbX is a putative phosphatase that is suggested to revert the stressosome to a nonstressed conformation after primary stress by removing phosphate groups from RsbR1 and RsbS poststress (31, 32). The role of RsbX in nonstressed conditions (to prevent SigB activation) is less clear, and a better understanding of its role and regulation is thus needed. The *sigB* and the *rsbX* genes lie adjacent to each within the same operon (*rsbV-rsbW-sigB-rsbX*), whose transcription is σ^B^ dependent, suggesting a potential functional connection between the gene products (Fig. 1).

In this work, we examined the function of the putative RsbX phosphatase in modulating the activity of the stressosome and consequently regulating SigB activity and downstream processes. We show that RsbX is crucial to reduce SigB activation levels under nonstressed conditions. In the absence of RsbX, increased SigB activity levels cause decreased biofilm formation as well as mobility. The motility phenotype of the *rsbX* mutant arises through decreased expression of the major flagellin protein FlaA. We also show that a strain lacking RsbX survives low pH better than the wild type by continuously overexpressing SigB-regulated genes. Surprisingly, in a strain lacking RsbX, we show that the phosphorylation pattern of the RsbR1 protein in the stressosome is only modestly changed, even though our results could suggest that the stressosome composition in this strain is rather affected. Our results highlight a crucial role for RsbX during both stress and nonstress conditions in downregulating SigB-controlled genes, resulting in major physiological changes to the bacterium.

## RESULTS

### RsbX suppresses SigB activity at nonstressed conditions.

We have previously identified a fitness disadvantage of a Δ*rsbX* mutant and a fitness advantage of a Δ*sigB* mutant strain compared to the wild-type strain. After 5 days of growth in culture (∼30 generations), bacteria lacking RsbX were outcompeted by wild-type bacteria despite being in a 1,000:1 excess at the onset of the experiment (30). Absence of SigB, on the other hand, outcompeted the wild-type strain at the same conditions. In B. subtilis, RsbX resets the stressosome after stress and is important to maintain the stressosome in a “sensing-ready” mode (23, 32). A similar phenotype has been suggested in L. monocytogenes where RsbX was shown to downregulate SigB activity poststress and in stationary phase (31). To further examine the role of RsbX in L. monocytogenes, a Δ*rsbX* knockout mutant was constructed. Also, a Δ*sigB* Δ*rsbX* double mutant was constructed to differentiate the effects exerted by RsbX and SigB, respectively. In addition, a plasmid carrying an IPTG (isopropyl-β-d-thiogalactopyranoside)-inducible copy of *rsbX* was constructed and incorporated into the chromosome of the Δ*rsbX* mutant strain.

**FIG 2:**
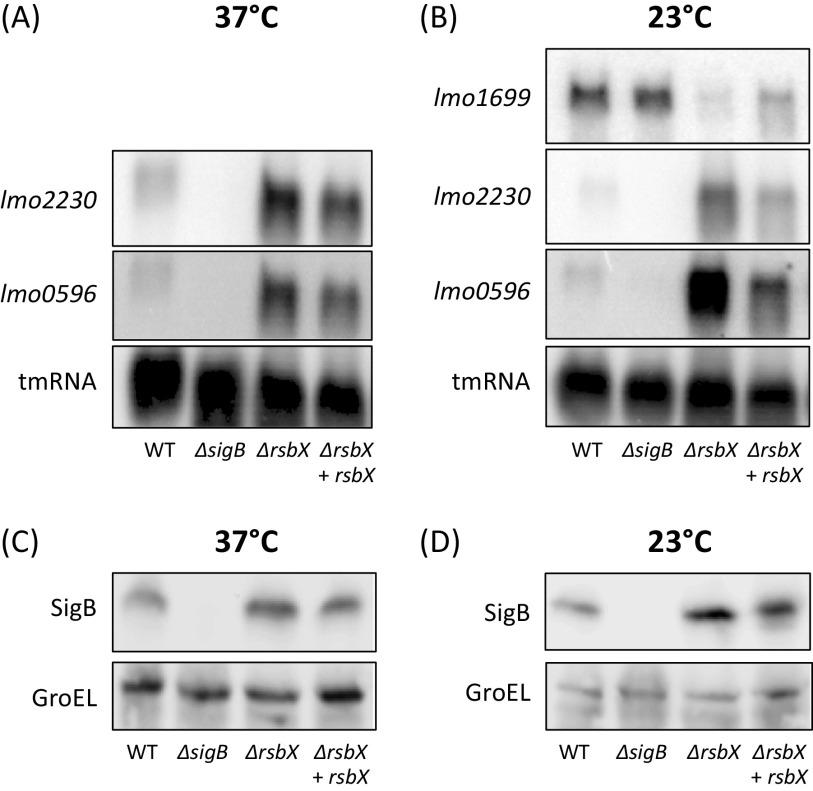
(A and B) Expression of SigB-regulated genes in different genetic backgrounds. Northern blot analysis showing expression levels of positively (*lmo2230* and *lmo0596*) and negatively (*lmo1699*) SigB-regulated genes. The strains (WT, Δ*sigB*, Δ*rsbX*, and Δ*rsbX* + *rsbX*) were grown at 37°C (A) or 23°C (B) in BHI medium in darkness to prevent light-induced stress, with constant agitation (180 rpm). Samples were taken when cultures reached an OD_600_ of ∼0.8, and the RNA was extracted. tmRNA was used as a loading control. *n* = 3. (C and D) Western blot analysis determining levels of SigB. WT, Δ*sigB*, Δ*rsbX*, and Δ*rsbX* + *rsbX* strains were grown in BHI at 37°C (C) or 23°C (D) in BHI medium in darkness with constant agitation until OD_600_ of ∼0.8 was reached. Samples were taken and protein extracted before Western blot analysis using anti-SigB antibodies. GroEL levels were used as a loading control; *n* = 3.

We were interested to examine if RsbX is needed to maintain low levels of SigB activity under nonstressed conditions in the EGD-e strain background. As a readout for SigB activity, we monitored the transcription of two strongly SigB-dependent genes, *lmo2230*, encoding a putative arsenate reductase, and *lmo0596*, encoding a putative *trans*-membrane protein with an unknown function (33, 34). Also, we examined expression of *lmo1699*, encoding a chemotaxis protein which is only expressed at low temperatures and negatively regulated by SigB (35). Since light induces SigB activity through the blue light receptor RsbL (4, 15–17), we incubated bacteria at 23 or 37°C in nonstressed (dark, flasks wrapped with aluminum foil and grown in water bath) and stressed (light, flasks grown in water bath without aluminum foil) conditions. RNA was isolated from bacteria grown to mid-log phase (optical density at 600 nm [OD_600_], ∼0.8) before being subject to Northern blot analysis. *lmo2230* and *lmo0596* transcription was induced under nonstressed (dark) conditions in the Δ*rsbX* mutant compared to the wild type at both 23 and 37°C (Fig. 2A and B). This effect could partially be suppressed by expressing *rsbX* in a Δ*rsbX* mutant. The role of RsbX in stressed (light) conditions was more limited; expression of *lmo2230* and *lmo0596* was similar in the wild type and the Δ*rsbX* mutant when the bacteria were exposed to light (see Fig. S1 in the supplemental material). In line with the expression data observed for *lmo2230* and *lmo0596*, expression of the SigB-repressed *lmo1699* was greatly reduced in the Δ*rsbX* mutant at both stress and nonstressed conditions compared to the wild type (Fig. 2B; Fig. S1). Surprisingly, the expression pattern of *lmo1699* in the wild type was similar at both light and dark conditions, suggesting that other regulatory pathways contribute to *lmo1699* expression at nonstressed conditions.

Since *sigB* and *rsbX* lie adjacent to each other on the SigB-activated *rsbVWsigBrsbX* transcript (Fig. 1), it was possible that the increased expression of *lmo2230* and *lmo0596* and the reduced expression of *lmo1699* observed in the Δ*rsbX* mutant were caused by an effect on the stability of the transcript rather than on the activity of SigB *per se*. Xia and coworkers indeed observed an increased SigB level in a strain lacking RsbX, but only following bacterial stress (31). Since we observed an increased transcription of SigB-regulated genes under nonstressed conditions, we monitored the SigB protein expression in different strain backgrounds grown in dark (nonstressed) and compared it to light (stressed) conditions at 23°C and 37°C, respectively. At 37°C, the levels of SigB did not significantly increase in the Δ*rsbX* mutant compared to the wild type (Fig. 2C; Fig. S2). However, at 23°C, the level of SigB was significantly increased in the Δ*rsbX* mutant compared to the wild type at light conditions (Fig. 2D; Fig. S2). This indicates that the increased levels of SigB might contribute to the elevated expression of SigB-regulated genes at lower temperatures, but not at higher temperatures, where it is likely that the activity of SigB is elevated instead. The increased SigB levels are likely caused by the positive feedback regulation by SigB of the p*rsbVWSigBrsbX* operon (Fig. 1) (31).

**FIG 3:**
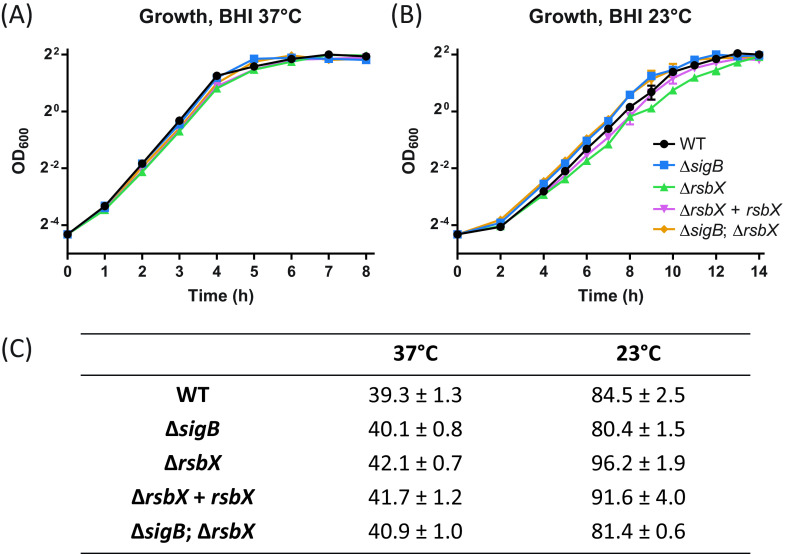
(A and B) Growth curves of WT, Δ*sigB*, Δ*rsbX*, Δ*rsbX* + *rsbX*, and Δ*sigB* Δ*rsbX* strains. Bacteria were grown in BHI with constant agitation of 180 rpm at either 37°C (A) or 23°C (B) until stationary phase was reached. Bacterial growth was determined by OD_600_ (*n* = 3). (C) Generation times derived from panels A and B in minutes of each indicated strain along with standard deviations.

The growth rate of the Δ*rsbX* mutant was similar to the growth rate observed for the wild type at 37°C in brain heart infusion (BHI) medium (Fig. 3). At 23°C, we observed a lower growth rate of the Δ*rsbX* mutant compared to the wild type (Fig. 3). As stated above, we have previously shown that the overall fitness of the Δ*rsbX* mutant is dramatically reduced when cocultured with the wild type, indicating that the strain lacking RsbX has a fitness disadvantage (30). A Δ*sigB* mutant and a Δ*sigB* Δ*rsbX* double mutant grew slightly faster than the wild type at lower temperatures, indicating that the reduced growth rate observed in the Δ*rsbX* mutant was due to an increased activation level of SigB and presumably an elevated expression of SigB-activated genes. Altogether, our data indicate that RsbX continuously suppresses SigB activity levels under nonstressed conditions.

### RsbX and SigB have opposite effects when bacteria are exposed to lethal acid stress.

**FIG 4:**
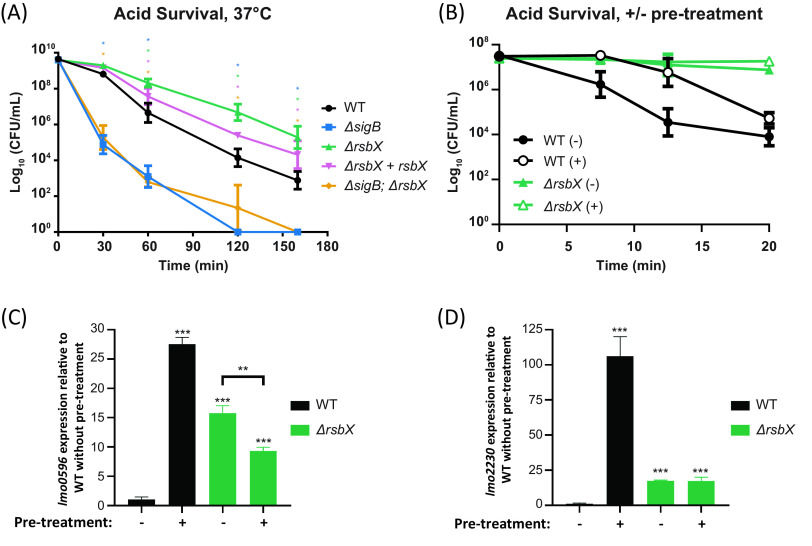
(A) Acid survival assay. WT, Δ*sigB*, Δ*rsbX*, Δ*rsbX* + *rsbX*, and Δ*sigB* Δ*rsbX* strains were grown overnight and resuspended in BHI of pH 2.5 and incubated at 37°C. Samples were taken at indicated time points and spread on agar plates to determine survival rate (CFU per milliliter). The graphic shows the average values of 3 biological replicates. A 2-way ANOVA with multiple comparisons was used for statistical analysis comparing all strains at the different time points with WT. *, *P* < 0.05. (B) Acid adaptation assay at 37°C. WT and the Δ*rsbX* mutant were grown until OD_600_ of ∼0.4 was reached when the cultures were either acidified (+) or not (−) at pH 5.0. (B) After 15 min, the cultures were acidified to pH 2.5 and the CFU per milliliter was counted at indicated time points. (C and D) The total RNA was extracted after 20 min of mild acidification, and the levels of transcripts of *lmo2230* (C) and *lmo0596* (D) were measured using RT-qPCR and normalized against 16S rRNA. Student's *t* test was used for statistical analysis. **, *P* < 0.01; **, *P* < 0.001 (*n* = 3).

It has previously been shown that strains lacking SigB are more stress susceptible (36–38). We therefore asked whether a strain lacking RsbX (and hence showing an increased SigB activity level) would have a benefit if the bacterium encountered a hazardous environment. To test this, bacteria were exposed to a lethal pH of 2.5. The absence of RsbX significantly increased bacterial survival compared to wild type when bacteria were exposed to pH 2.5 at 37°C, whereas survival was reduced in the Δ*sigB* mutant and the Δ*sigB* Δ*rsbX* double mutant by 10^2^- to 10^3^-fold compared to wild type (Fig. 4A). We observed no difference in survival for the Δ*rsbX* mutant at 23°C compared to wild type whereas bacterial survival in the Δ*sigB* mutant and the Δ*sigB* Δ*rsbX* double mutant were reduced up to 10^8^-fold at 23°C (Fig. S3). This suggests that the increased survival of the Δ*rsbX* mutant at low pH was due to an upregulated expression of SigB-regulated genes, making the bacterium initially more prepared for lethal environments. To investigate whether a mild stress adaptation would affect bacterial survival at pH 2.5, bacteria were preexposed to pH 5.0 before lowering the pH to 2.5. While the wild type displayed a clear adaptive response with increased acid resistance following pretreatment at pH 5.0, the Δ*rsbX* mutant displayed an intrinsically higher acid resistance without pretreatment, probably due to higher SigB activity at 37°C (Fig. 4B). To examine whether SigB activity was different in strains with or without adaption (preexposed to pH 5.0), RNA was isolated from wild type or the Δ*rsbX* mutant strains before performing reverse transcriptase quantitative PCR (RT-qPCR) to determine the levels of *lmo2230* and *lmo0596* at 37°C (Fig. 4C and D). As expected, the Δ*rsbX* mutant showed an increased SigB activity compared to the wild type in the absence of stress adaptation and did not show an induced *lmo2230* and *lmo0596* expression upon stress. Instead, strains lacking RsbX had a similar level of SigB activity regardless of stress adaptation or not. Surprisingly, after mild stress adaptation, the Δ*rsbX* mutant had lower *lmo2230* and *lmo0596* expression than the wild type (Fig. 4C and D).

Together, these data suggest that the Δ*rsbX* mutant continuously expresses SigB-activated genes, which help the strain in surviving unexpected stresses but impair its competitive growth index (Fig. 2 to 4; Fig. S1 to S3) (30). They further suggest that the transduction of mild acid stress signals into the SigB activation pathway requires a functional RsbX protein.

### RsbX is important for biofilm formation and motility.

**FIG 5:**
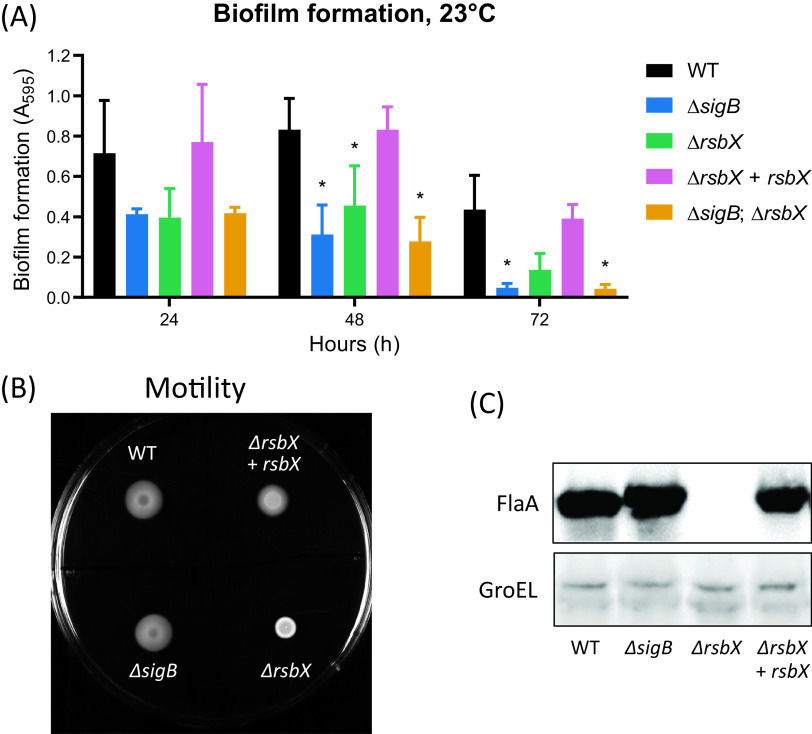
(A) Biofilm production of WT, Δ*sigB*, Δ*rsbX*, Δ*rsbX* + *rsbX*, and Δ*sigB* Δ*rsbX* strains. Bacteria were statically grown in a 96-well round-bottomed plate for 24 h, 48 h, and 72 h at 23°C in TSB medium. The graphics show the average values of 3 biological replicates. A 2-way ANOVA with multiple comparisons was used for statistical analysis comparing all strains at the different time points with WT. *, *P* < 0.05; *n* = 3. (B) Motility assay. WT, Δ*sigB*, Δ*rsbX*, and Δ*rsbX* + *rsbX* strains were spotted on a motility agar plate (BHI; 0.3% agar) and grown at bench conditions (∼23°C) for 24 h. *n* = 3. (C) Levels of FlaA in different genetic backgrounds. WT, Δ*rsbX*, and Δ*rsbX* + *rsbX* strains were grown in BHI at 23°C with constant agitation (180 rpm) until an OD_600_ of ∼0.8 was reached. At this time point, protein was extracted and the levels of FlaA determined by Western blotting, using anti-FlaA antibodies. GroEL was used as a loading control. *n* = 3.

L. monocytogenes is able to form biofilms (e.g., multicellular communities), and SigB has previously been shown to be important for its proper formation (39, 40). We therefore tested the biofilm formation capabilities in different strains. The amount of biofilm formed at 23°C was decreased in the Δ*rsbX* mutant compared to the wild type (Fig. 5A). Surprisingly, the Δ*sigB* mutant and the Δ*sigB* Δ*rsbX* double mutant behaved as the Δ*rsbX* mutant, suggesting that maximal biofilm formation requires a fine-tuned expression of SigB-regulated genes. It could also indicate a role for RsbX in biofilm regulation independent of the stressosome complex. At 37°C, the Δ*rsbX* mutant formed less biofilm compared to the wild type, whereas the Δ*sigB* and the Δ*sigB rsbX* mutants instead formed more biofilm (Fig. S4).

L. monocytogenes lacking the motility apparatus has been shown to have a decreased biofilm formation and vice versa; decreased biofilm formation is often associated with reduced motility (40, 41). In contrast to many other bacterial species, L. monocytogenes is motile only at lower temperatures (<30°C). When we examined bacterial motility at low temperatures (23°C), the Δ*rsbX* mutant showed a decreased motility compared to the wild type (Fig. 5B). However, the Δ*rsbX* mutant had an unusual appearance on the motility agar plate; the bacteria formed a dense clump at the point of the inoculum and did not spread horizontally on the agar surface. In contrast, the Δ*sigB* mutant and the Δ*sigB* Δ*rsbX* double mutant showed a motility phenotype similar to the wild type. Since the absence of the major flagellar subunit FlaA abolishes motility, we sought to examine whether FlaA expression was altered in the nonmotile Δ*rsbX* mutant. Indeed, we were unable to detect FlaA in the Δ*rsbX* mutant strain compared to the wild type, suggesting that RsbX positively controls FlaA expression, directly or indirectly (Fig. 5C). A Δ*sigBs* Δ*rsbX* double mutant showed a similar pattern of motility and FlaA expression as the Δ*sigB* mutant, suggesting that the motility phenotype observed in a Δ*rsbX* mutant is caused by an increased SigB activity level (Fig. S4).

### RsbX only slightly modulates RsbR1 phosphorylation.

**FIG 6:**
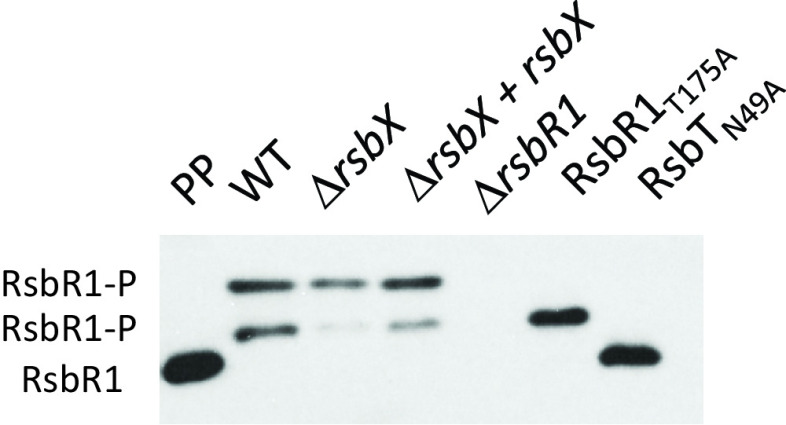
Phos-tag separation followed by Western blotting to determine phosphorylation of RsbR1 in different genetic backgrounds. WT, Δ*rsbX*, Δ*rsbX* + *rsbX*, Δ*rsbR1*, RsbR1_T175A_, and RsbT_N49A_ strains were grown in BHI at 37°C until an OD_600_ of ∼0.4 was reached. At this time point, protein was extracted, and the migration and level of RsbR1 were determined by Western blotting using anti-RsbR1 antibodies. Purified His-tagged protein was used as a positive control, the Δ*rsbR1* mutant was used as a RsbR1 negative control, the RsbR1_T175A_ mutant is lacking one phosphorylation site, and the RsbT_N49A_ mutant is a kinase mutant, abolishing RsbR1 phosphorylation. *n* = 3.

Stressosome-mediated activation of SigB requires a series of RsbT-mediated phosphorylation events of RsbS and RsbR1 (23, 32). RsbR1 becomes phosphorylated at positions T175 and T209 through the kinase activity of RsbT (14, 29). The phosphorylation pattern of RsbR1 can be monitored by using Phos-tag gels, which allow separation of different protein isoforms depending on their phosphorylation status. Since absence of RsbX increased SigB activity levels, we speculated that the Δ*rsbX* mutant would show an altered phosphorylation pattern compared to the wild type. A strain expressing RsbR1_T175A_, which lacks one phosphorylation site, was used as a control together with a strain expressing a kinase-deficient RsbT protein (RsbT_N49A_). Somewhat surprisingly, our results indicate that absence of RsbX only slightly modulated the phosphorylation pattern of cytosolic RsbR1 compared to the wild type (Fig. 6). In contrast to the expected role of a phosphatase, we did not observe an increased level of phosphorylated RsbR1 in the Δ*rsbX* mutant. The middle band, which likely represents the monophosphorylated form of RsbR1 on residue T209 (since it was the only band present in the T175A mutant background), was instead present at a reduced level in the Δ*rsbX* mutant (Fig. 6).

### RsbX is important to maintain stressosome multiprotein composition.

**FIG 7:**
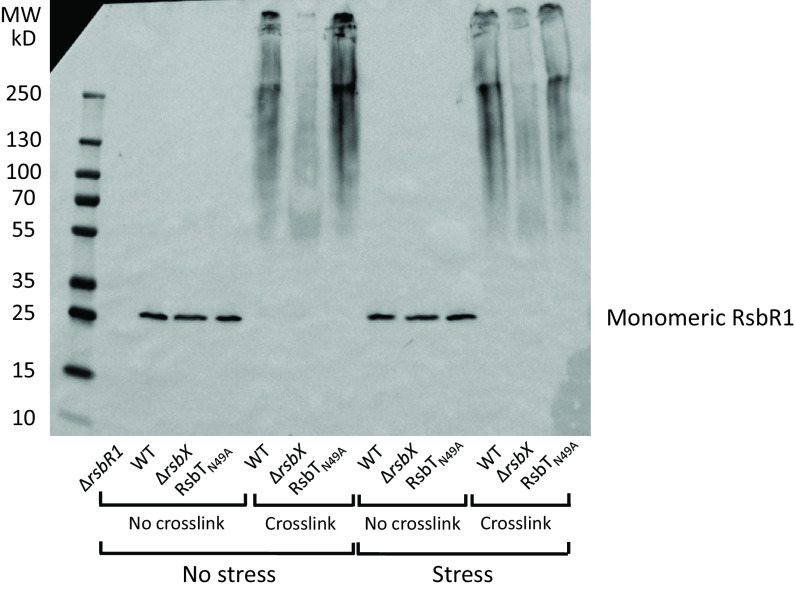
*In vivo* RsbR1 cross-linking experiment. WT, Δ*rsbX*, and RsbT_N49A_ (N49A) strains were grown in BHI at 37°C until an OD_600_ of 0.8 was reached when the cultures were either stressed by 0.5 M NaCl for 5 min or not. At this time point, samples were either cross-linked by formaldehyde for 10 min or not, after which protein was extracted and Western blot analysis performed using an anti-RsbR1 antibody. A Δ*rsbR1* mutant was used as a negative control. *n* = 3.

Another prediction of the stressosome-mediated activation of SigB is a change in the composition of the stressosome upon its activation by stress, culminating with RsbT being released (23, 42). To monitor such putative structural changes, we performed an *in vivo* cross-linking experiment using formaldehyde followed by Western blotting to detect RsbR1. A large, slowly migrating RsbR1 complex possibly constituting a stressosome(s) was observed in the wild type at both nonstressed and stressed (0.5 M NaCl) conditions at 37°C (Fig. 7). In contrast, the absence of RsbX dramatically changed the appearance of the RsbR1 signal, with almost no higher-molecular-weight complexes detectable. This could indicate that RsbX prevents RsbR1 repositioning in a wild-type background. We also used the RsbT_N49A_-expressing strain as a control since this strain is unable to phosphorylate RsbR1 under any condition and likely would prevent changes in stressosome composition. As for the wild type, a slowly migrating RsbR1 complex was observed in the RsbT_N49A_ mutant (Fig. 7). When examining the migration of cross-linked RsbT, we observed a decreased appearance of high-molecular-weight RsbT complexes in a Δ*rsbX* mutant compared to the wild type and Rsb_TN49A_ strains, although the effect was not as strong as observed for RsbR1 (Fig. S5).

Curiously, the overall RsbR1 Western blot signal in the cross-linked preparations decreased in the Δ*rsbX* mutant compared to the wild type. Since the RsbR1 levels were similar between the WT and the Δ*rsbX* mutant (Fig. 7, non-cross-linked samples), the reduced amount of high-molecular-weight RsbR1 in the Δ*rsbX* mutant could be due to reduced levels of the RsbR1 protein complexes entering the gel in a Δ*rsbX* mutant. When heating the samples to reverse the formaldehyde cross-linking, this hypothesis was strengthened (Fig. S6): with an increasing time of heating, the amount of the monomeric form of RsbR1 and a band corresponding to an RsbR1 dimer were increased compared to the nonheated sample. These data could suggest that the activity of RsbX on other stressosome components is necessary to maintain the normal quaternary structure of the stressosome.

## DISCUSSION

In this paper, we examined whether the putative RsbX phosphatase was important to maintain the stressosome in a conformation preventing SigB activation, also in conditions of no stress. In B. subtilis, it has been shown that RsbX indeed acts as a phosphatase, removing phosphate groups from RsbR and RsbS (22, 32, 43). Surprisingly, therefore, we were unable to detect an increased level of RsbR1 phosphorylation in a strain lacking RsbX in L. monocytogenes. Whereas phosphorylation of RsbR_T209_ was reduced in the Δ*rsbX* mutant, no accompanying increase in phosphorylation could be observed at any other residue (i.e., T175) of RsbR1. Although RsbX*_Bs_* and RsbX*_Lm_* share approximately 30% identity and 67% similarity, our results may indicate a role for RsbX other than being a phosphatase. Despite not having a big effect on the phosphorylation pattern of RsbR1, absence of RsbX dramatically altered the migration of cross-linked RsbR1 samples on native gels followed by Western blotting. It could be suggested that the large, undefined high-molecular-weight RsbR1 signal detected in the cross-linked wild type would constitute stressosome complexes. Similar but less dramatic mobility changes were observed for cross-linked RsbT in presence or absence of RsbX. Since RsbT and not RsbR1 is predicted to be released from the stressosome upon stress activation, our data are a bit unanticipated (23, 42). It should, though, be noted that we cannot exclude the possibilities that the complex is unable to access the gels in the Δ*rsbX* mutant or that the antibodies are unable to recognize the RsbR1 and RsbT proteins in the same strain. Clearly, further experiments are required to determine the mechanism by which RsbX functions in L. monocytogenes and how it might control stressosome architecture.

Our finding that RsbX is important at both stressed and nonstressed conditions is in line with previous findings in B. subtilis determining a role for RsbX in downregulating SigB activity (23, 43). Based on the data presented here and in earlier studies, we speculate that the role of RsbX in L. monocytogenes might be to keep the stressosome in a dormant, stress sensing-proficient conformation under conditions where the ambient stress levels are low. A stronger stress leads to a phosphorylation cascade, inevitably leading to RsbT dissociation from the stressosome and, eventually, SigB activation. Here, RsbX would be needed to reset the stressosome to an inactivated state, although the mechanism underpinning this process in L. monocytogenes needs to be determined. It should, though, be noted that transcription of the *rsbVW-sigB-rsbX* operon is positively regulated by SigB. Hence, an increase in SigB activity upon stress could also result in an increased level of SigB. Indeed, the SigB protein levels were increased 2-fold in the Δ*rsbX* mutant, but only at 23°C, compared to the wild-type strain. Such increased levels of SigB protein could at least partially explain the elevated expression of SigB-regulated genes observed in a strain lacking RsbX.

Induced SigB activity in the Δ*rsbX* mutant also slowed down bacterial growth slightly at low temperatures and decreased the competitiveness of the bacterium (30). On the other hand, absence of RsbX also made the bacterium more resilient to acid stress. The basal SigB activity (without stress pretreatment) was as expected elevated in a strain lacking RsbX compared to the wild type. Surprisingly, the Δ*rsbX* mutant was unable to reach the same SigB activity, even after stress pretreatment (Fig. 4C and D). This could indicate that RsbX, by some yet-unknown mechanism, is important for effective transduction of stress signals through the stressosome.

The strongest phenotype we observed associated with RsbX was its importance for motility. Absence of RsbX almost completely abolished motility and FlaA expression. The mechanism lying behind this phenotype remains unknown but should include an RsbX-dependent upregulation of motility gene expression through downregulation of SigB activity. A role for SigB in L. monocytogenes motility gene expression has previously been put forward, and exposure to blue light decreases motility through RsbL (15, 16, 44, 45). The SigB-mediated motility repression is at least partially mediated by a SigB-dependent expression of the motility repressor MogR, as well as an antisense RNA (46). We have observed that the expression of several other motility genes is decreased in the Δ*rsbX* mutant (T. Tiensuu et al., unpublished data), suggesting a role for RsbX in regulation of motility gene expression. Since expression of *lmo1699* (encoding a chemotaxis protein) is reduced in the Δ*rsbX* mutant, it could indicate that L. monocytogenes has adopted a dedicated strategy through SigB to repress expression of motility genes. Whether this is mediated through MogR/antisense RNA remains to be elucidated.

A previous study showed a role for RsbX in the recovery phase following a primary stress but did not identify any growth or survival differences between the wild type and the Δ*rsbX* mutant at prestress or mild stress conditions (31). In contrast to that study, we were unable to observe a decreased survival of a Δ*rsbX* mutant exposed to low pH, a condition where, instead, we observed an elevated survival of the Δ*rsbX* mutant. It is not known why the results differ between the studies, but it should be noted that the strain background used by Xia et al. was different from ours (10403S versus EGD-e). Another explanation could be that we maintained bacteria in darkness at nonstressed conditions since light is a strong stress inducer in L. monocytogenes (4, 15–17).

**FIG 8:**
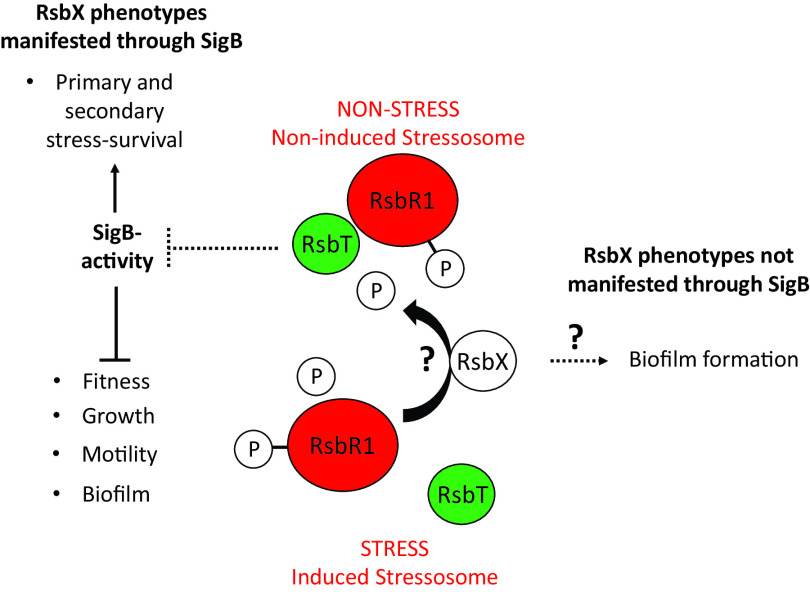
Schematic model showing the function and phenotypes associated with RsbX. RsbX is required to prevent SigB activation at nonstressed conditions, thereby maximizing the fitness of the bacteria, motility, and biofilm formation. However, at conditions of stress, the SigB pathway is essential for bacterial survival. Exactly how RsbX interacts with the stressosome is not clear, and RsbX might play a role in biofilm formation independent of SigB.

With this work, we conclude that RsbX indeed can act as a “gatekeeper” to avoid unnecessary activation of the SigB pathway at nonstressed conditions to save energy, thereby maximizing fitness (modeled in Fig. 8) (31). At conditions of stress, SigB needs to be activated for the bacteria to survive. The exact mechanism by which RsbX interacts with the stressosome and preserves it in an inactive conformation requires further work.

## MATERIALS AND METHODS

### Bacterial strains, plasmids, and growth conditions.

Bacterial strains and plasmids used in this study are listed in Table 1. Listeria monocytogenes strains were mainly grown in BHI (BD Bacto) broth or agar, either at 37°C or 23°C. To induce *rsbX* expression in the Δ*rsbX rsbX* strain, 1 mM IPTG (isopropyl-β-d-1-thiogalactopyranoside; final concentration) was added to the culture.

**TABLE 1:** Strain and plasmids used in this study

Strain or plasmid	Reference or source
L. monocytogenes strains	
EGD-e (WT)	55
EGD-e Δ*sigB*	46
EGD-e Δ*rsbX*	30
EGD-e Δ*rsbX* + *rsbX*	This study
EGD-e Δ*sigB* Δ*rsbX*	This study
EGD-e Δ*rsbR1*	14
EGD-e RsbR1_T175A_	14
EGD-e RsbT_N49A_	14
Plasmids	
pIMK3	47
pMAD	49
p*rsbX*	This study

### Construction of genetically modified L. monocytogenes.

Oligonucleotides used in this study are listed in Table 2. For the complementation of the Δ*rsbX* mutant strain, plasmid p*rsbX* was constructed. Briefly, a PCR fragment (using the primers Comp *rsbX* fwd and Comp *rsbX* rev) was digested with the indicated restriction endonucleases (Table 2) and subcloned into the corresponding restriction sites of the IPTG-inducible plasmid, pIMK3 (47). The plasmid construct was verified by endonuclease digestion and further by sequencing and was used to transform the L. monocytogenes EGD-e Δ*rsbX* mutant strain by conjugation, obtaining the Δ*rsbX* + *rsbX* mutant strain (48).

**TABLE 2:** Oligonucleotides used in this study

Oligonucleotide sequence (5′–3′)	Target
Cloning	
GGGGGATCCGGGGAGATCGATGCTTATACAGCG	*sigB*, *rsbX* a fwd (BamHI restriction site underlined)
GGGGTCGACTTTATCAGGTTGAGATACTTTTGGC	*sigB*, *rsbX* b rev (SalI restriction site underlined)
GGGGTCGACTAACCATAACACCTTTTAAACTAGCG	*sigB*, *rsbX* c fwd (SalI restriction site underlined)
GGGCCATGGCGTAACCACAAGTCCAATAAGTGG	*sigB*, *rsbX* d rev (NcoI restriction site underlined)
AGTCCCAATATAATCATTTATCAACTC	pMAD_seq F
TGGTCGTCATCTACCTG	pMAD_seq R
GGGGGATCCTGAGGAAGTGGAGTAAATG	Comp *rsbX* fwd (BamHI restriction site underlined)
GGGCTGCAGTGTTCTCACACCCAATCTG	Comp *rsbX* rev (PstI restriction site underlined)
Northern blotting	
CAATGAGATCAGCAT	*lmo2230* L
GCATATTCGAAGTGC	*lmo2230* U
CCTCCACATTAATGTTTGG	*lmo0596* fwd
GCAAGAAACCTAGTAAGATGG	*lmo0596* rev
CGCAACTCTCCTTCTCGATGG	*lmo1699* fwd
GCTTTATAGCTCGCACTTTGG	*lmo1699* rev
CCTCGTTATCAACGTCAAAGCC	tmRNA fwd
CGGCACTTAAATATCTACGAGC	tmRNA rev
RT-qPCR	
CATATTCGAAGTGCCATTGC	lmo2230_F
CTGAACTAGGTGAATAAGACAAAC	lmo2230_R
GGGTACTAGCTGACGGAATTTTATC	lmo0596_F
CCCACATACCGAAAAGTAATACGAG	lmo0596_R

The L. monocytogenes EGD-e Δ*sigB* Δ*rsbX* mutant strain was constructed by PCR amplification of approximately 800-bp flanks of both *sigB* and *rsbX*, respectively. One of the fragments was digested and cloned into the pMAD vector (pMAD::Δ*sigB*) (49). The second fragment amplified was also digested and cloned into pMAD::Δ*sigB*, creating pMAD::Δ*sigB* Δ*rsbX*. Confirmation of the construct was carried out by endonuclease digestion and sequencing, using primers pMAD_seq F and pMAD_seq R, and, once confirmed, it was transformed into electrocompetent L. monocytogenes EGD-e by electroporation. Allelic replacement was performed according to reference 49, with some minor alterations. Transformants were selected at 30°C on BHI plates containing erythromycin (5 μg/ml), and isolated colonies were grown at 39°C overnight in BHI with erythromycin (5 μg/ml). To obtain integrants, serial dilutions were plated on BHI with erythromycin (5 μg/ml) plus X-Gal (5-bromo-4-chloro-3-indolyl-β-d-galactopyranoside; 50 μg/ml) plates and grown at 41°C overnight. Isolated blue colonies were selected and grown at 30°C in BHI overnight, followed by incubation at 39°C for 3 h. Serial dilutions were plated on LA X-Gal (50 μg/ml) plates and grown at 30°C for 2 days. White colonies (indicating excision and loss of plasmid) were screened for erythromycin sensitivity, and deletion of *sigB* and *rsbX* was confirmed by PCR.

### Growth rate determination.

Listeria monocytogenes cultures were grown in BHI media at 37°C for 16 h (overnight). The overnight cultures were diluted to an OD_600_ of 0.05 and incubated at the indicated temperature (37°C or 23°C) in a water bath with constant agitation (∼180 rpm). The OD_600_ was measured once per hour until the cultures reached stationary phase. The growth curves were performed in biological triplicates, and the data were analyzed using GraphPad Prism (version 7.0).

### Biofilm production assay.

Determination of biofilm production was performed essentially as described in reference 50. Listeria monocytogenes cultures were grown overnight in tryptic soy broth (TSB) medium at 37°C and then diluted 1:500 in fresh TSB medium and again incubated overnight at 37°C. The culture was diluted a second time 1:20 in TSB and aliquots of 100 μl transferred to a 96-well round-bottom sterile plate. These plates were incubated for 24 h, 48 h, and 72 h, either at 23°C or 37°C, with static growth at bench light conditions. After the corresponding incubation time, the cultures were aspirated from the wells before addition of 150 μl of sterile water and subsequent aspiration to remove loosely associated bacteria. This was repeated twice. The plates were dried at 37°C for 45 min. An aqueous 1% crystal violet solution was prepared and aliquoted (150 μl) into the plate’s wells and again incubated at 37°C for 45 min. The crystal violet solution was aspirated from the wells, followed by 3 washes with 150 μl of sterile water, and the pellet was air dried at 37°C for 30 min. A 95% ethanol solution was added to destain each individual well and determine the concentration of crystal violet by measuring the absorbance at 595 nm in a plate reader (LabSystems Multiskan RC). A total of 3 biological replicates were performed. The data were analyzed in GraphPad Prism (version 7.0).

### Acid survival assay.

Overnight cultures of L. monocytogenes were grown for 16 h at either 23°C or 37°C before addition of 1 mM IPTG for 1.5 h. The stationary-phase cultures were pelleted by centrifugation and resuspended in BHI medium previously acidified to pH 2.5. Resuspensions were incubated in a water bath at either 37°C or 23°C for 160 min and 420 min, respectively. Samples were taken at indicated time points (0, 30, 60, 120, and 160 min at 37°C; 0, 120, 180, 240, 300, 360 and 420 min at 23°C). Each sample was serially diluted to 1 × 10^−7^ and plated onto BHI agar plates. The plates were incubated at 37°C for 24 h. Colonies from all three biological replicates were counted, and CFU per milliter was determined and plotted. A 2-way analysis of variance (ANOVA) test with multiple comparisons was performed on the data, with a confidence interval of 95%. The data analysis and the statistical analysis were done using GraphPad Prism (version 7.0).

For the acid adaptation experiment, L. monocytogenes wild-type (WT) and Δ*rsbX* strains were grown and subject to acid shock. Briefly, cultures were grown at 37°C at constant shaking of 150 rpm for 16 h until stationary phase and diluted to an initial OD_600_ of 0.05 in fresh BHI. Bacteria were allowed to grow until mid-log phase (OD_600_, 0.4). One set of the bacterial cultures was acidified to pH 5, whereas the pH in the control cultures was unaltered. Both sets were incubated for 15 min at 37°C and diluted 1:10 in BHI acidified to pH 2.5. Samples were taken at 0, 7.5, 12.5, and 20 min, respectively, serial diluted in phosphate-buffered saline (PBS) (Sigma) from 10^−2^ to 10^−6^, and plated on BHI agar. Plates were incubated at 37°C for 24 h, and the CFU per milliliter was calculated from the resulting colony counts, performed in triplicate. Statistical analysis was performed using Student's *t* test.

### RT-qPCR.

For RT-qPCR analysis of *lmo2230* and *lmo0596* expression after acid adaptation, L. monocytogenes EGD-e and the Δ*rsbX* mutant strains were diluted in RNAlater (Sigma). RNA was extracted using RNeasy minikit (Qiagen) according to the manufacturer’s recommendations. Cells were lysed by bead beating in a FastPrep-24 at a speed of 6 m/s for 40 s twice with a 5-min interval when samples were kept on ice. The DNA present in the samples was digested with Turbo DNA-free kit (Invitrogen) according to the manufacturer’s recommendations. The integrity of the RNA was verified by electrophoresis in 0.7% (wt/vol) agarose gels. The synthetises of cDNA was performed using SuperScript III first-strand synthesis system (Invitrogen) according to the manufacturer’s recommendations. The cDNA obtained was quantified using NanoDrop 2000c (Thermo Scientific) and diluted to a final concentration of 7 ng/ml. RT-qPCR was performed using a Quanti-Tect SYBR green PCR kit (Qiagen) and primers for the target genes *lmo2230* and *lmo0596* and the normalization control 16S rRNA (Table 2). Samples were analyzed on a LightCycler 480 system (Roche), cycle quantification was performed by using LightCycler 480 software version 1.5.1 (Roche), and the relative expression formula was applied (51, 52). 16S rRNA expression was used as a reference housekeeping gene. Results were converted to log_2_ relative expression normalized for the expression of the nontreated L. monocytogenes wild type (*n* = 3). Statistical analysis was performed using Student's *t* test.

### Motility plate assay.

Listeria monocytogenes cultures were grown in BHI media at 37°C for approximately 16 h. An aliquot of 2 μl was spotted on a BHI motility agar plates (BHI plus 0.3% [wt/vol] agar) and grown for 24 h at laboratory temperature (∼23°C). After 24 h of growth, a photograph of the plate was taken.

### RNA isolation for Northern blotting.

Isolation of RNA was performed essentially as previously described in reference 56 with some minor changes. L. monocytogenes strains were grown overnight at 37°C before being diluted 1:100 in BHI medium. The cultures were grown in water baths with constant agitation (180 rpm) at either 37°C or 23°C until an OD_600_ of ∼0.8 was reached. For dark conditions, cultures were wrapped in aluminum foil. At that point, 0.2× volume of a 5% phenol/95% ethanol solution was added and the samples collected and pelleted by centrifugation at 4°C and 6,000 × *g* for 10 min. The pellets were kept at −80°C. The samples were resuspended in a resuspension solution (10% glucose, 12.5 mM Tris-HCl [pH 7.5], and 5 mM EDTA) and transferred to a tube containing 0.4 g of glass beads and 0.5 ml acid phenol-chloroform. The bacteria were disrupted using the FastPrep machine, and the mix was centrifuged for 5 min at 16,800 × *g* and 4°C; the aqueous phase added to 1 ml of Tri reagent, and the reaction was incubated for 5 min at room temperature. The aqueous phase (top) was transferred to a new tube, 100 μl of chloroform was added, and the mix was centrifuged. After centrifugation, one more chloroform extraction was performed before precipitating the RNA by isopropanol and freezing the samples at −20°C for 30 min. The pellet washed with 75% ethanol and resuspended in 180 μl of diethyl pyrocarbonate (DEPC)-treated water. To remove any DNA, the samples were subject to a DNase treatment (20 U) and incubated 45 min at 37°C before addition of phenol-chloroform/indole-3-acetic acid (IAA) (Ambion) and centrifugation (5 min, 16,800 × *g*, 4°C). The aqueous phase was extracted with chloroform and centrifuged. The RNA pellet was precipitated in 1:10 volume of 3 M sodium acetate (NaAc) (pH 4.6) and 2.5 volumes of 99.9% ethanol, incubated at −20°C for 30 to 60 min, and centrifuged for 20 min. The pellet was air dried for 5 min and resuspended in 200 μl DEPC-treated water. The extracted RNA was analyzed on a 1.2% agarose gel to verify transcript integrity. The concentration of the RNA was measured on a NanoDrop 1000 machine.

### Northern blotting.

Northern blotting was performed using a protocol based on Tiensuu et al. (16) with minor alterations. Twenty micrograms of isolated RNA were precipitated overnight at −20°C in 1:10 of the volume of 3 M NaAc (pH 4.6) and 2.5 volumes of 99.9% ethanol, followed by a centrifugation step of 30 min, 16,800 × *g*, at 4°C. The pellet was resuspended in 15 μl of RNA sample buffer (100 μl DEPC-treated water, 50 μl 10× HEPES, 250 μl formamide, and 100 μl formaldehyde) and denatured at 65°C for 3 min, followed by an incubation on ice. We added 6× formamide dye (95% dionized formamide, 10 mM EDTA, 0.1% [wt/vol] bromophenol blue, 0.1% [wt/vol] xylene cyanol, and 0.1% [wt/vol] orange G), and the samples were loaded onto a 1.2% agarose gel containing 1× HEPES buffer (10× HEPES buffer includes 0.2 M HEPES, 50 mM NaAc, and 10 mM EDTA, adjusted to pH 7) and 7.3% formaldehyde. The gel was run in 1× HEPES for 4 h at 100 V. The RNA was transferred to a Hybond-N membrane (Amersham) by capillary transfer in 20× buffer SSC. The membranes were cross-linked by UV light, prehybridized in Rapid hyb buffer (GE Healthcare UK Limited) for about 2 h at 60°C and then hybridized with ^32^P-dATP α-labeled DNA fragments using Prime-a-Gene labeling system at 60°C overnight. DNA fragments were amplified by PCR using corresponding primers for *lmo2230*, *lmo0596*, and *lmo1699* and transfer-messenger RNA (tmRNA). Membranes were washed (0.5% SDS and 2× SSC [1× SSC is 0.15 M NaCl plus 0.015 M sodium citrate] at room temperature for 15 min followed by 0.5% SDS and 0.1× SSC at 60°C for 15 min), exposed in a phosphorimager cassette, and developed using the Typhoon FLA 9500 scanner (GE Healthcare). A total of 3 biological replicates were performed. Quantification was done with ImageQuant 8.2, and analysis was done on GraphPad Prism (version 7.0).

### Protein extraction.

To detect FlaA levels, bacterial strains were grown overnight at 37°C in BHI with 1 mM IPTG. The cultures were diluted to an OD_600_ of 0.05 and grown at 23°C and 175 rpm in BHI plus 1 mM IPTG for 24 h. After 24 h, the OD_600_ was measured, and 5 ml was collected from the cultures and centrifuged for 10 min at 7,800 × *g*. For SigB samples, the bacteria were grown overnight at 37°C in BHI, diluted 1:100 into fresh BHI plus 1mM IPTG, and grown at both 37°C and 23°C, either in dark (cultures wrapped in aluminum foil) or light conditions, until an OD_600_ of 0.8 was reached. Afterward, 12 ml of culture were collected and centrifuged (10 min, 7,800 × *g*, 4°C). The pellets were kept at −80°C until the protocol was resumed.

For the extraction of protein, essentially the protocol by Fliss et al. was followed (53) with minor alterations. Pellets were resuspended in 1 ml SET buffer (50 mM NaCl, 5 mM EDTA, and 30 mM Tris-HCl, pH 8). Samples were centrifuged for 10 min at 7,800 × *g*, and the pellet was resuspended in 0.5 ml of acetone, kept on ice for 10 min, and centrifuged as described above. Pellets were completely resuspended in 200 μl of a mixture containing 50 mM Tris-HCl, pH 6.5, 200 U/ml mutanolysin (Sigma-Aldrich), 200 U/ml DNase I (Roche), and 0.1 mg/ml RNase A and incubated for 30 min at 37°C. We added 4× SDS-PAGE protein sample buffer to a final concentration of 1×, and the samples were boiled at 95°C for 15 min. The loading of the samples was corrected based on their OD_600_ or adjusted based on a Coomassie staining gel. The samples were then separated on a 12% SDS-polyacrylamide gel or a 4 to 20% Mini-Protean TGX precast protein gels (catalog no. 4561096; Bio-Rad) and transferred to a nitrocellulose membrane, and a Western blot analysis was performed with appropriate antibodies.

### Western blotting and Phos-tag detection.

Protein samples were boiled 15 min at 95°C and then loaded onto a 12% SDS-PAGE gel (SigB and FlaA samples) or a 4 to 20% Mini-Protean TGX precast protein gels (Bio-Rad) and run in 0.25 M Tris, 1.92 M glycine, and 1% SDS (80 V for 20 min plus 200 V for 1 h more). Separated proteins were transferred to a nitrocellulose membrane using a semidry transfer system (Trans-Blot Turbo transfer system; Bio-Rad) for 30 min at 25 V. The membrane was blocked with 5% (wt/vol) skimmed milk in 1% PBS with Tween (PBS-T) for 1 h followed by 3 washes with PBS-T and incubated with the primary antibody (RsbR1, 1:500 dilution; RsbT, 1:500; SigB, 1:10,000 [a kind gift of T. Hain]; FlaA, 1:5,000 [a kind gift of Stanley Wall]; and GroEL, 1:5,000) overnight at 4°C with agitation. The membranes were washed 3 times with PBS-T and incubated with the secondary antibody (horseradish peroxidase [HRP]-conjugated goat anti-rabbit secondary antibodies; catalog no. as09602; Agrisera, Vännäs, Sweden) for 1 h at room temperature with agitation in a dilution of 1:10,000. An ECL Prime Western blotting system (Amersham/GE Healthcare) was used for the detection, and the membrane was exposed and visualized in ImageQuant.

SuperSep Phos-tag gels (Fujifilm) are precast polyacrylamide gels containing a Phos-tag molecule with a Zn^2+^ ion. This functional molecule binds specifically to the phosphate group, trapping phosphorylated proteins during SDS-PAGE, and allowing the separation of phosphorylated and nonphosphorylated proteins, detected as different bands in the gel. After sample preparation (as above), samples were separated for 15 min at 80 V and 95 min at 140 V. A pretreatment of gels before transfer was required to remove zinc ions, consisting of 3× 20-min washes in 1× transfer buffer (250 mM Tris, 0.192 M glycine, 10% [vol/vol] methanol, and 10 mM EDTA) followed by 1 wash with transfer buffer lacking EDTA for 10 min. The remaining procedure was as described above.

### *In vivo* cross-linking.

The protocol for *in vivo* protein cross-linking was based on the protocol (54) with some modifications. The cultures were grown overnight at 37°C, diluted 1:100, and grown in darkness (cultures wrapped in aluminum foil) at 37°C until an OD_600_ of 0.8 was reached. Afterwards, 0.5 M NaCl was added to half the cultures and incubated for 5 min. For the cross-linking, formaldehyde was added to the cultures to a final concentration of 0.74% (vol/vol) and incubated for an extra 10 min with agitation. To quench the reaction, 1:10 of the volume of an ice-cold and sterile glycine solution (0.125 M final concentration) in PBS was added. Thereafter, 12 ml were collected and pelleted by centrifugation (10 min, 7,800 × *g*, 4°C), whereafter the protocol for protein extraction was followed. The samples (either nonboiled to avoid reversion of cross-linking or boiled to reverse the cross-linking) were loaded onto a 4 to 20% Mini-Protean TGX precast protein gels; Bio-Rad) before Western blotting using RsbR1 or RsbT (1:500 dilution) primary antibodies.

### Protein extraction and subcellular fractionation.

Subcellular fractionation was performed as previously described (14, 29), with some minor alterations. Cultures were grown overnight at 37°C, diluted 1:100 in 50 ml of BHI and grown at 37°C with agitation until an OD_600_ of ∼0.4 was reached. The cultures were then boiled for 20 s and centrifuged (4°C for 18 min at 10,000 × *g*), and the pellet was washed in 9 ml PBS. The pellets were kept at −80°C until the protocol was resumed, and the pellets were resuspended in 1 ml of TS buffer (10 mM Tris-HCl [pH 6.9], 10 mM MgCl_2_, and 0.5 M sucrose) and centrifuged (10,000 × *g*, 18 min, 4°C). Pellets were resuspended in 1.2 ml of a lysis buffer (TS buffer containing 60 μg/ml mutanolysin, 250 μg/ml RNase A, and protease inhibitor cocktail [PI]; Roche Diagnostics) and incubated for 5 h at 37°C with slow-rotating agitation. The protoplasts were recovered by centrifugation (15,000 × *g*, 10 min, 4°C), the supernatant was discarded (cell wall fraction), and the pellet was washed with 1 ml PBS and centrifuged (10,000 × *g*, 18 min, 4°C). The protoplasts were resuspended in 500 μl of 50 mM Tris (pH 7.0), 10 μl 50× IP (protease inhibitor cocktail), and 1 μg/ml DNase and lysed by sonication (20 s, 3 times, 0.7 level of intensity, constant of 0.5). Unbroken cells were removed by centrifugation (20,000 × *g*, 10 min at 4°C). The supernatant underwent 2 rounds of ultracentrifugation (rotor 100.3, Optima TLX ultracentrifuge, 100,000 × *g*, 1 h at 4°C). After the first centrifugation step, the supernatant obtained corresponded to the cytosol fraction, and after the second centrifugation, the pellet was resuspended in 500 μl 50 mM Tris, pH 7.0, corresponding to the membrane fraction.
