# Supplementary material for: Listeria monocytogenes Requires the RsbX Protein To Prevent SigB Activation under Nonstressed Conditions
Source: J Bacteriol. 2022 Jan 18;204(1):e00486-21. doi: 10.1128/JB.00486-21 (PMC8765406; doi:10.1128/JB.00486-21)
Supplement: Supplemental file 1 — Fig. S1 to S6. Download JB.00486-21-s0001.pdf, PDF file, 1.4 MB [file jb.00486-21-s0001.pdf]

Supplementary material

Listeria monocytogenes requires the RsbX protein to prevent SigB-activation under non-stressed conditions.

Ana H. Oliveira[1,2,3], Teresa Tiensuu[1,2,3], Duarte N. Guerreiro[4], Hasan Tükenmez[1,2,3,5], Charlotte Dessaux[6,¤], Francisco García-del Portillo[6], Conor O'Byrne[4] and Jörgen Johansson[1,2,3,*].

[1]Laboratory for Molecular Infection Medicine Sweden, Umeå University, Umeå, Sweden

[2]Department of Molecular Biology, Umeå University, Umeå, Sweden

[3]Umeå Centre of Microbial Research, Umeå University, Umeå, Sweden

[4]Bacterial Stress Response Group, Microbiology, School of Natural Sciences, National University of Ireland, Galway, Ireland

[5]Department of Chemistry, Umeå University, Sweden

[6]Laboratory of Intracellular Bacterial Pathogens, National Center of Biotechnology, (CNB)-CSIC, Spain

*To whom correspondence should be addressed: jorgen.johansson@umu.se

Supplementary figure legends

Figure S1. (A, B) Expression of SigB regulated genes in different genetic backgrounds. Northern blot analysis showing expression levels of positively (*lmo2230* and *lmo0596*) and negatively (*lmo1699*) SigB regulated genes. The strains (WT, Δ*sigB*, Δ*rsbX* and Δ*rsbX*+*rsbX*) were grown at 37°C (A) or 23°C (B), in BHI medium in light conditions, with constant agitation

(180 r.p.m.). Samples were taken when cultures reached OD_{600} nm~0.8 when RNA was extracted. tmRNA was used as a loading control. n=3. (C, D) Western blot of determining levels of SigB. WT, Δ*sigB*, Δ*rsbX* and Δ*rsbX* + *rsbX* strains were grown in BHI, at 37°C (C) or 23°C (D) in BHI medium in light conditions with constant agitation until OD_{600} nm~0,8 was reached. Samples were taken and protein extracted before Western blot analysis using anti-SigB antibodies. GroEL levels were used as a loading control. n=3.

Figure S2. Relative levels of SigB expression in different genetic backgrounds quantified from Figure 2C and D and Figure S1C and D. The amount of SigB was quantified and normalized to the expression of GroEL in indicated strains. Dunnett's multiple comparisons test was used for statistical analysis. * reflects a *p* value of < 0.05 and ** a *p* value of < 0.01. ns = non significant.

Figure S3. Acid survival assay. WT, Δ*sigB*, Δ*rsbX*, Δ*rsbX* + *rsbX* and Δ*sigB;* Δ*rsbX* strains were grown overnight and resuspended in BHI of pH 2.5, and incubated at 23°C. Samples were taken at indicated time points, spread on agar-plates to determine survival rate (CFU/ml). The graphic shows the average values of 3 biological replicates. A 2-way ANOVA with Multiple comparisons was used for statistical analysis comparing all strains at the different time points with WT. * reflects a *p* value < 0.05.

Figure S4. (A) Biofilm production of WT, Δ*sigB*, Δ*rsbX,* Δ*rsbX* + *rsbX* and Δ*sigB;* Δ*rsbX* strains. Bacteria were statically grown in a 96-well round-bottomed plate, for 24h, 48h and 72h, at 23°C in TSB medium. The graphics show the average values of 3 biological replicates. A 2-way ANOVA with Multiple comparisons was used for statistical analysis comparing all strains at the different time points with WT. * reflects a P value<0.05. (B) Motility assay. WT, Δ*sigB*,

Δ*rsbX* and Δ*sigB;* Δ*rsbX* strains were spotted on a motility agar plate (BHI, 0,3% agar) and grown at bench conditions (~23°C) for 24 hours. n=3. (C) Levels of FlaA in different genetic backgrounds. WT, Δ*sigB*, Δ*rsbX*, Δ*rsbX* + *rsbX and* Δ*sigB;* Δ*rsbX* strains were grown in BHI, at 23°C, with constant agitation (180 r.p.m.) until OD_{600} nm~0.8 was reached. At this time-point, protein was extracted and the levels of FlaA determined by western blot analysis, using anti-FlaA antibodies. A commassie-stained gel was used as a loading control (below). n=3.

Figure S5. *In vivo* RsbT crosslinking experiment. WT, Δ*rsbX* and $RsbT_{N49A}$ (kinase mutant) mutant strains were grown in BHI, at 37°C, until OD_{600} of 0.8 was reached. At this OD, half of the cultures were stressed by 0.5M of NaCl for 5 min. For samples being cross-linked, formaldehyde was added and incubated for 10 mins before protein extraction and western blot using an anti-RsbT antibody. A Δ*rsbR1* mutant strain (also devoid of RsbT expression) was used as a negative control. n=3.

Figure S6. *In vivo* RsbR1 cross-linking and cross-linking reversal. WT, Δ*rsbX* and $RsbT_{N49A}$ strains were grown in BHI, at 37°C, until OD_{600} nm of 0.8. For samples being cross-linked, formaldehyde was added and incubated for 10 minutes. For the reversal of the cross-linking, the same samples were incubated at 95°C, for either 15 minutes (crosslink 15 min) or 30 minutes (crosslink 30 min). At these time-points, protein was extracted and western blot performed using an anti-RsbR1 antibody. The monomeric form of RsbR1 (~30 kD) is indicated by * and the putative dimeric form of RsbR1 (~70kDa) is indicated by **. A Δ*rsbR1* mutant was used as a negative control.

Fig. S1

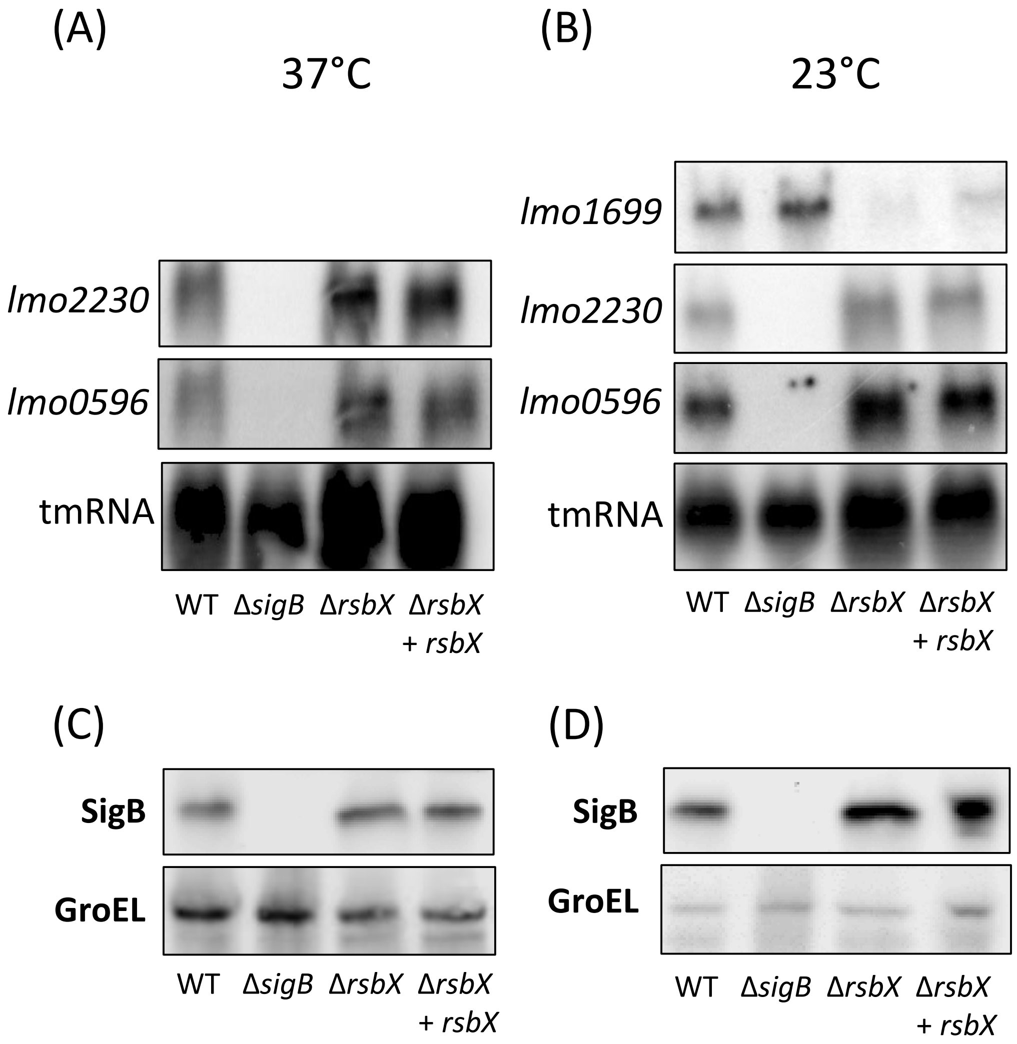
(A)
37°C
lmo2230
lmo0596
tmRNA
WT ΔsigB ΔrsbX ΔrsbX + rsbX
(B)
23°C
lmo1699
lmo2230
lmo0596
tmRNA
WT ΔsigB ΔrsbX ΔrsbX + rsbX
(C)
SigB
GroEL
WT ΔsigB ΔrsbX ΔrsbX + rsbX
(D)
SigB
GroEL
WT ΔsigB ΔrsbX ΔrsbX + rsbX

Fig. S2

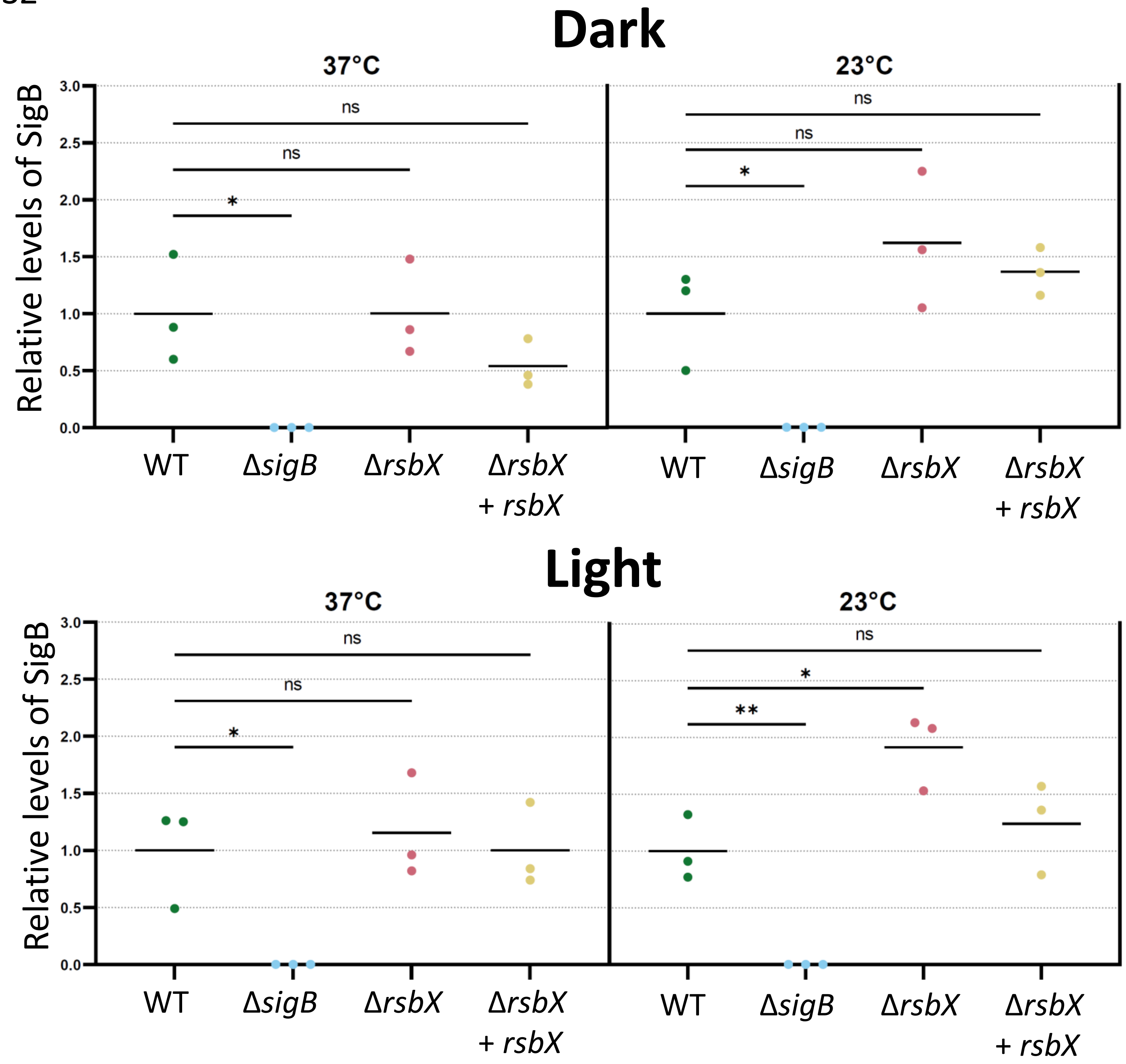
Dark
37°C
23°C
Relative levels of SigB
3.0
2.5
2.0
1.5
1.0
0.5
0.0
ns
ns
*
ns
ns
*
WT
ΔsigB
ΔrsbX
ΔrsbX + rsbX
WT
ΔsigB
ΔrsbX
ΔrsbX + rsbX
Light
37°C
23°C
Relative levels of SigB
3.0
2.5
2.0
1.5
1.0
0.5
0.0
ns
ns
*
ns
*
**
WT
ΔsigB
ΔrsbX
ΔrsbX + rsbX
WT
ΔsigB
ΔrsbX
ΔrsbX + rsbX

Fig. S3

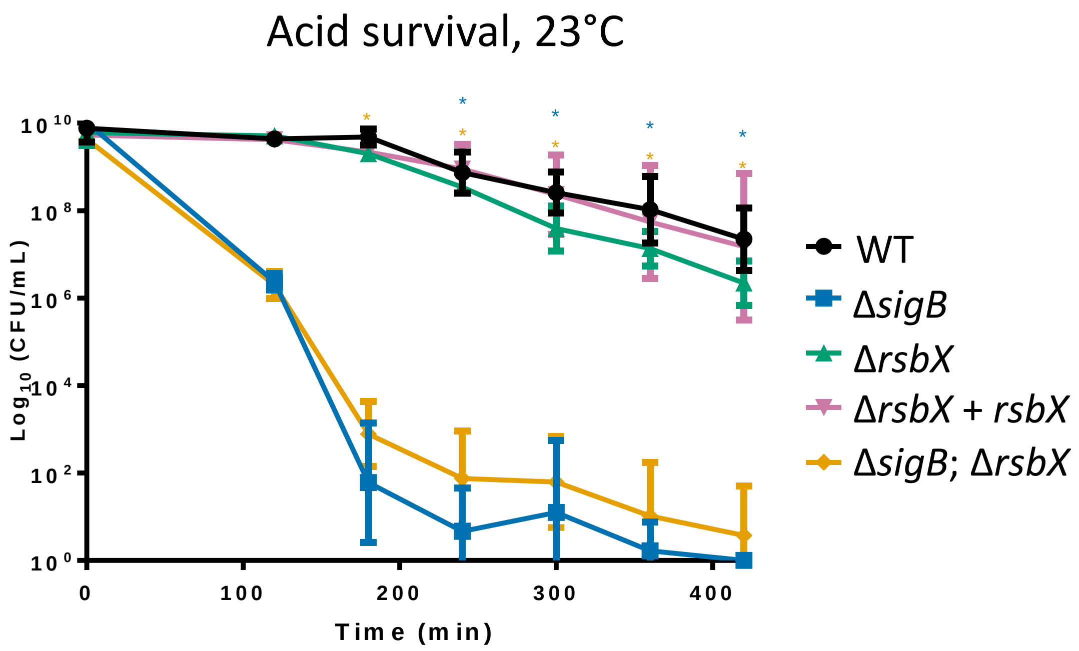
Acid survival, 23°C
10^10
10^8
10^6
10^4
10^2
10^0
Log10 (CFU/mL)
0
100
200
300
400
Time (min)
WT
ΔsigB
ΔrsbX
ΔrsbX + rsbX
ΔsigB; ΔrsbX

Fig. S4

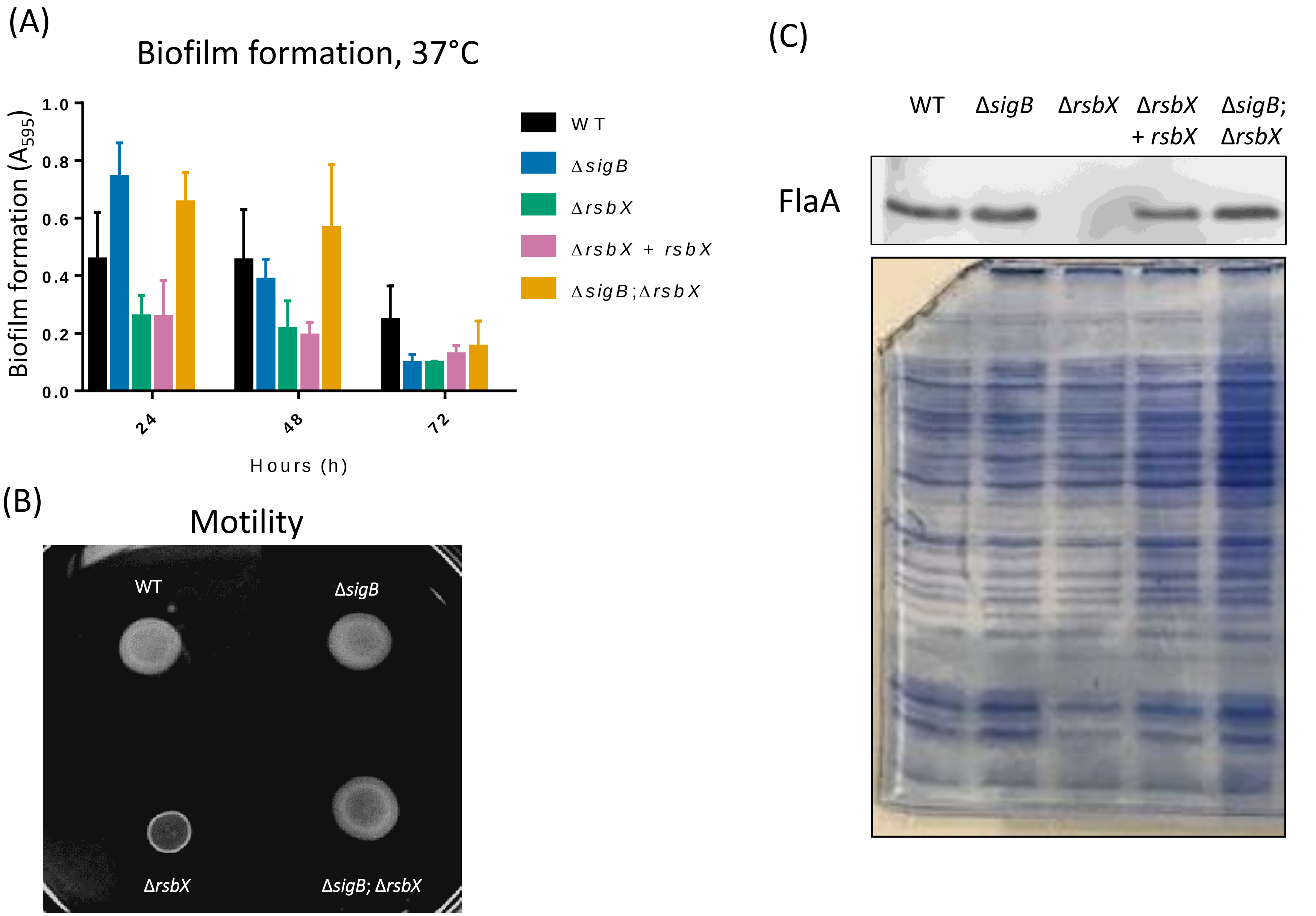
(A)
Biofilm formation, 37°C
Biofilm formation (A595)
1.0
0.8
0.6
0.4
0.2
0.0
24
48
72
Hours (h)
WT
ΔsigB
ΔrsbX
ΔrsbX + rsbX
ΔsigB;ΔrsbX
(B)
Motility
WT
ΔsigB
ΔrsbX
ΔsigB; ΔrsbX
(C)
WT
ΔsigB
ΔrsbX
ΔrsbX + rsbX
ΔsigB; ΔrsbX
FlaA

Fig. S5

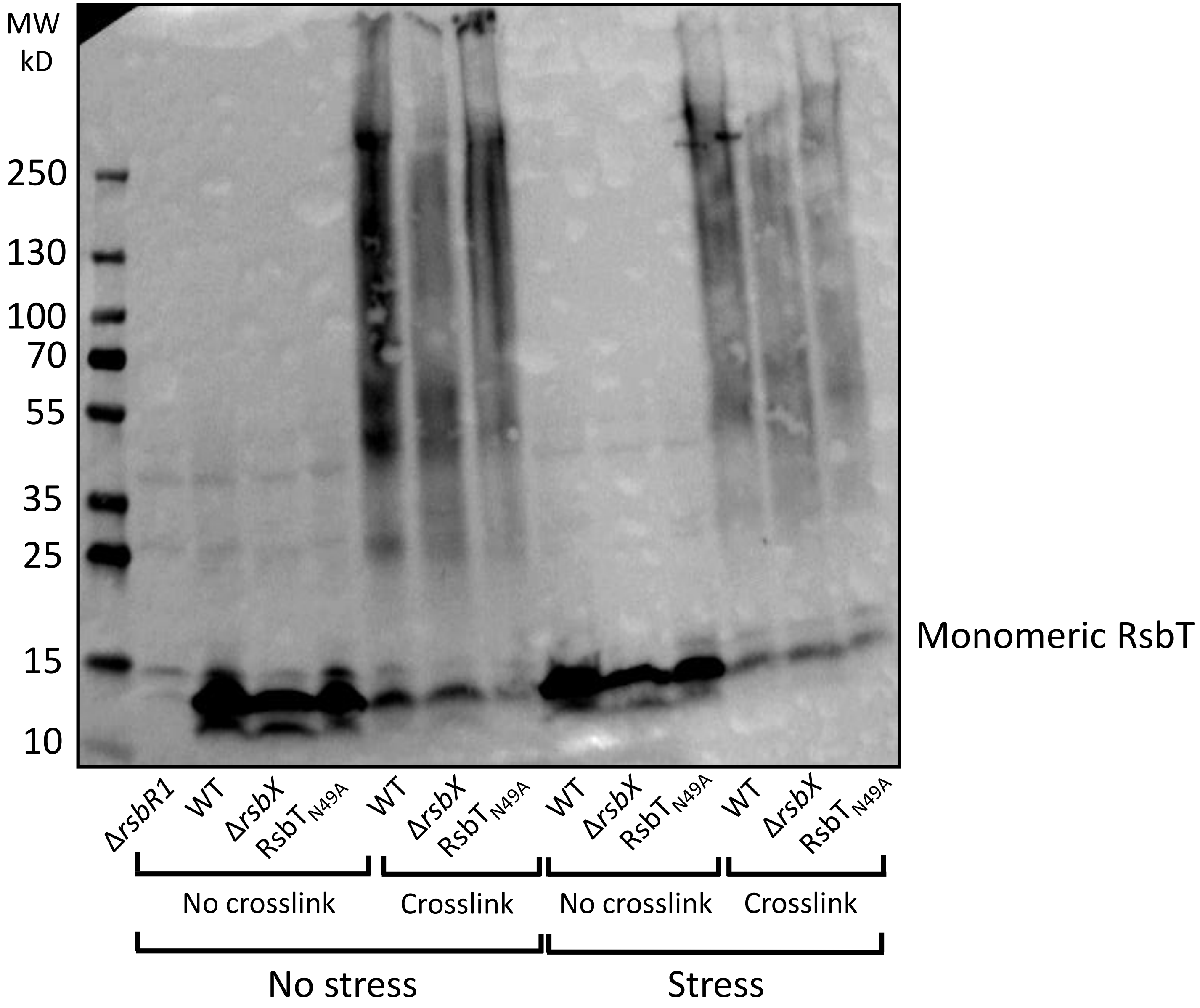
MW
kD
250
130
100
70
55
35
25
15
10
Monomeric RsbT
ΔrsbR1
WT
ΔrsbX
RsbT N49A
WT
ΔrsbX
RsbT N49A
WT
ΔrsbX
RsbT N49A
WT
ΔrsbX
RsbT N49A
No crosslink
Crosslink
No crosslink
Crosslink
No stress
Stress

Fig. S6

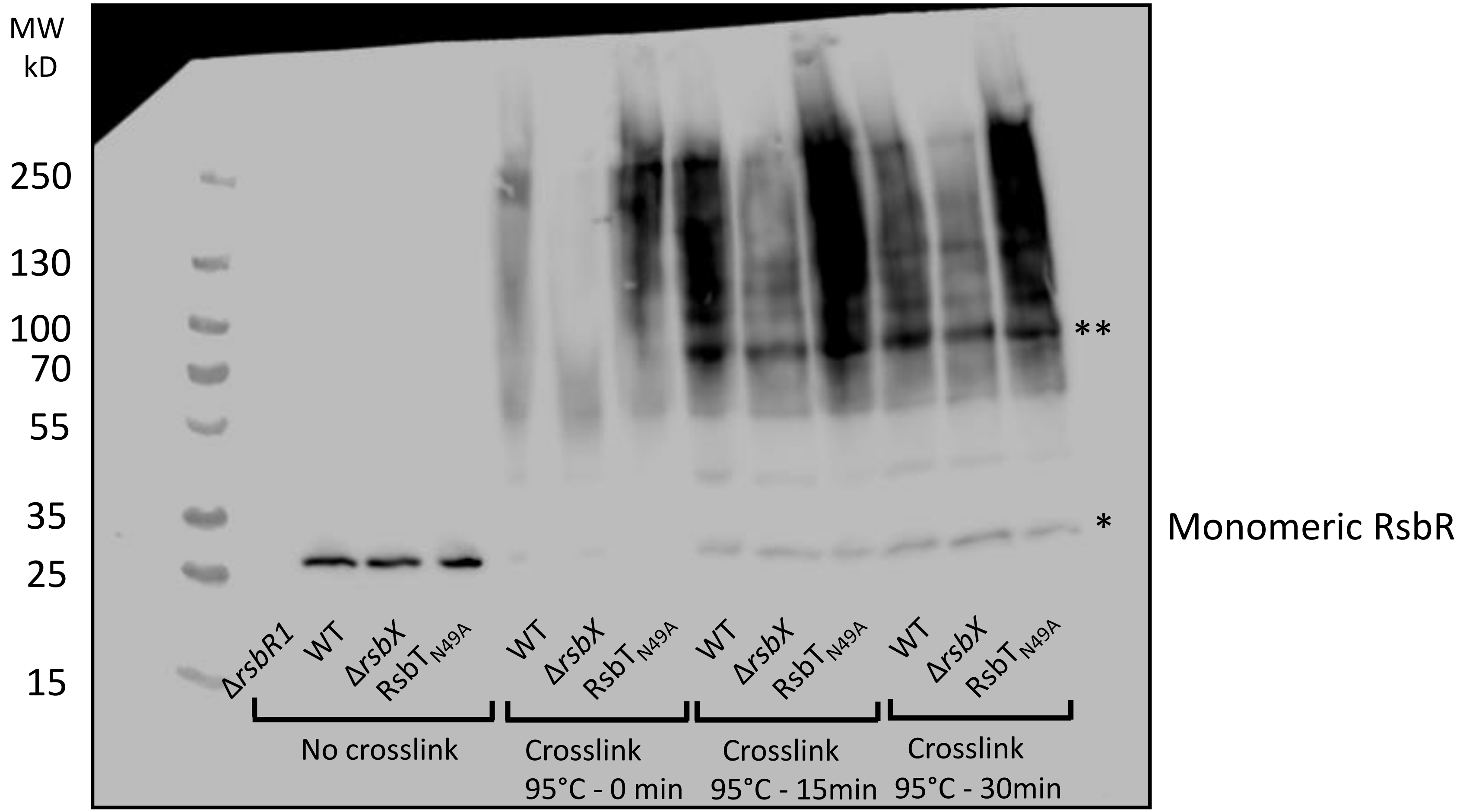
MW
kD
250
130
100
70
55
35
25
15
**
*
Monomeric RsbR
ΔrsbR1
WT
ΔrsbX
RsbT N49A
WT
ΔrsbX
RsbT N49A
WT
ΔrsbX
RsbT N49A
WT
ΔrsbX
RsbT N49A
No crosslink
Crosslink
95°C - 0 min
Crosslink
95°C - 15min
Crosslink
95°C - 30min
